# *BRCA1* and *BRCA2* Missense Variants of High and Low Clinical Significance Influence Lymphoblastoid Cell Line Post-Irradiation Gene Expression

**DOI:** 10.1371/journal.pgen.1000080

**Published:** 2008-05-23

**Authors:** Nic Waddell, Anette Ten Haaf, Anna Marsh, Julie Johnson, Logan C. Walker, kConFab Investigators, Milena Gongora, Melissa Brown, Piyush Grover, Mark Girolami, Sean Grimmond, Georgia Chenevix-Trench, Amanda B. Spurdle

**Affiliations:** 1 Queensland Institute of Medical Research, Brisbane, Australia; 2 Peter MacCallum Cancer Centre, Melbourne, Australia; 3 Institute for Molecular Biosciences, University of Queensland, Brisbane, Australia; 4 School of Molecular and Microbial Sciences, University of Queensland, Brisbane, Australia; 5 Department of Computer Science & Engineering, Indian Institute of Technology, Kharagpur, India; 6 Department of Computing Science, University of Glasgow, Glasgow, United Kingdom; University of Pennsylvania, United States of America

## Abstract

The functional consequences of missense variants in disease genes are difficult to predict. We assessed if gene expression profiles could distinguish between *BRCA1* or *BRCA2* pathogenic truncating and missense mutation carriers and familial breast cancer cases whose disease was not attributable to *BRCA1* or *BRCA2* mutations (*BRCAX* cases). 72 cell lines from affected women in high-risk breast ovarian families were assayed after exposure to ionising irradiation, including 23 *BRCA1* carriers, 22 *BRCA2* carriers, and 27 *BRCAX* individuals. A subset of 10 *BRCAX* individuals carried rare *BRCA1/2* sequence variants considered to be of low clinical significance (LCS). *BRCA1* and *BRCA2* mutation carriers had similar expression profiles, with some subclustering of missense mutation carriers. The majority of *BRCAX* individuals formed a distinct cluster, but *BRCAX* individuals with LCS variants had expression profiles similar to *BRCA1/2* mutation carriers. Gaussian Process Classifier predicted *BRCA1*, *BRCA2* and *BRCAX* status, with a maximum of 62% accuracy, and prediction accuracy decreased with inclusion of *BRCAX* samples carrying an LCS variant, and inclusion of pathogenic missense carriers. Similarly, prediction of mutation status with gene lists derived using Support Vector Machines was good for *BRCAX* samples without an LCS variant (82–94%), poor for *BRCAX* with an LCS (40–50%), and improved for pathogenic *BRCA1/2* mutation carriers when the gene list used for prediction was appropriate to mutation effect being tested (71–100%). This study indicates that mutation effect, and presence of rare variants possibly associated with a low risk of cancer, must be considered in the development of array-based assays of variant pathogenicity.

## Author Summary

Inherited mutations in the genes *BRCA1* and *BRCA2* increase risk of breast cancer and contribute to a proportion of breast cancer families. However, more than half of the reported sequence alterations in *BRCA1* and *BRCA2* are currently of unknown clinical significance. We analysed gene expression in lymphoblastoid cell lines derived from blood of patients with sequence alterations in *BRCA1* and *BRCA2* and compared these to lymphoblastoid cells from familial breast cancer patients without such alterations. We then classified these lymphoblastoid cells based on their gene profiles. We found that *BRCA1* and *BRCA2* samples were more similar to each other than to familial breast cancer patients without *BRCA1/2* mutations, and that the type of sequence change in *BRCA1* and *BRCA2* (missense or truncating) influenced gene expression. We included in the study ten familial breast cancer samples, which carried sequence changes in *BRCA1* or *BRCA2*, that are believed to be of little clinical significance. Interestingly these samples were distinct from other familial breast cancer cases without any sequence alteration in *BRCA1* or *BRCA2*, indicating that further work needs to be performed to determine the possible association of these “low clinical significance” sequence changes with a low to moderate risk of cancer.

## Introduction

Approximately 7% of breast cancer cases occur in women with a strong family history of the disease [1]. Mutations in *BRCA1* and *BRCA2* account for a considerable proportion of these familial breast cancer cases, with the average cumulative risk in *BRCA1* and *BRCA2* mutation carriers by age 70 years estimated at 65% and 45%, respectively [2]. The Breast Cancer Information Core (BIC) database (http://research.nhgri.nih.gov/bic/) currently has more than 1400 and 1800 unique sequence variants listed in the *BRCA1* and *BRCA2* genes, respectively. These include frameshift, nonsense, missense, splice site alterations and polymorphisms. Greater than a third of the *BRCA1* and greater than half of the *BRCA2* unique variants are “unclassified variants” without compelling evidence of pathogenicity or functional significance. The majority of unclassified variants recorded in the BIC database are predicted missense changes (more than 400 *BRCA1* and 800 *BRCA2*). However other variants which may be categorised as unclassified variants are in-frame deletions or duplications, variants that may disrupt splicing, or variants in the 3′UTR that may affect RNA stability (www.kconfab.org). *BRCA1*/*2* unclassified variants represent a problem in the clinical setting as it is not known which variants are associated with the high risk of disease reported for classical truncating mutations.

Several functional assays may be used to determine the significance of unclassified variants, including transcription activation and complementation assays [3]–[9], but a disadvantage of biochemical assays is that they rely on the functions of specific domains of the protein, require specialized laboratory skills, and are time–consuming to perform. Other methods for classifying variants include the analysis of clinical and histopathological data [10], loss of heterozygosity analysis [11] and bioinformatic analysis to predict the effect of the amino acid change on structure and multiple sequence alignment strategies [12]; [13]–[15]. Integrated evaluation of unclassified variants which combines several approaches, such as the analysis of co-segregation of the mutation with disease, co-occurrence of the variant with a deleterious mutation, sequence conservation of the amino acid change, severity of amino acid change, tumor loss of heterozygosity, and tumor histopathology classification, provides a quantitative tool for the classification of variants [16]–[22]. This multifactorial method was developed to classify such rare unclassified variants into two categories, variants with features of classical high-risk mutations (termed pathogenic), and variants that do not have the features of a high-risk mutation (termed neutral or low clinical significance (LCS)). While the availability of appropriate biospecimens (e.g. number of families and tumors) for inclusion in likelihood prediction is a major factor determining the classification of any single variant, another major caveat of the multifactorial approach is that it is not appropriate for the evaluation of possible moderate or low risk variants, since it uses high-risk mutations as reference for the underlying assumptions [16],[19],[20]. Therefore, the current multifactorial method cannot exclude the possibility that rare variants classified to be of low clinical significance may be associated with a moderate or low risk of cancer.

Gene expression profiling has increased our understanding of the molecular events in breast tumor development, has been used to predict prognosis, and has characterised breast tumors into subtypes [23]–[27]. The value of expression profiling for identifying underlying high-risk gene mutation status is indicated by a number of studies. A distinct gene expression profile has been reported for breast tumors of *BRCA1* mutation carriers [23],[28],[29], expected to be homozygous for loss of BRCA1 function at the somatic level. In addition, the existence of distinct gene expression profiles for heterozygous loss of BRCA1 and BRCA2 function is supported by accurate separation of short-term cultures of fibroblasts carrying a germline mutation in the *BRCA1* or *BRCA2* genes, compared to healthy women undergoing reduction mammoplastic surgery with no family or personal history of any cancer or sporadic breast-cancer-affected controls [30],[31]. Lymphoblastoid cell lines (LCLs) have also been shown to have distinct mRNA expression phenotypes for heterozygous carriers of *ATM* mutations, some of which are known to be associated with an increased risk in breast cancer [32],[33]. These findings suggest that germline gene expression signatures, including those from fibroblasts or LCLs, may be used to define *BRCA1* or *BRCA2* mutation status and to assist in assessing the clinical significance of *BRCA1* and *BRCA2* unclassified variants.

In this study we compared LCL gene expression signatures of breast cancer cases carrying pathogenic mutations in *BRCA1* or *BRCA2,* to familial breast cancer cases with no known *BRCA1/2* mutations (*BRCAX*). We also considered the possibility that *BRCAX* individuals with a *BRCA1* or *BRCA2* sequence variant classified to be neutral/low clinical significance (LCS) using multifactorial likelihood analysis may differ in gene expression profile from *BRCAX* individuals without such sequence variants. In addition, since truncating alterations comprise the majority of known pathogenic mutations but most *BRCA1* and *BRCA2* unclassified variants are predicted missense alterations, we compared profiles from individuals with known missense or truncating mutations to determine if mutation effect will affect the mutation-associated expression profile for each gene. We derived gene lists to predict mutation status defined by gene and mutation effect, and then tested the efficacy of these gene lists to predict the gene mutation status of LCLs. We provide evidence that gene lists differ according to gene and mutation effect, and according to the presence of sequence variants of low clinical significance. We also demonstrate that the use of appropriately-derived gene lists improves the prediction of pathogenicity of known mutations.

## Results

### Differences in LCL Post-Irradiation Gene Expression between *BRCAX* Individuals with or without a Sequence Variant of Low Clinical Significance

**Figure 1:**
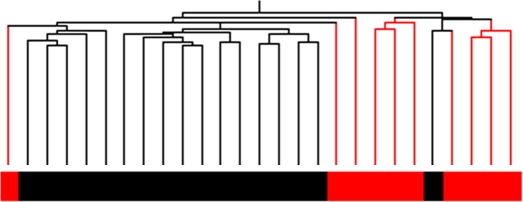
Unsupervised Hierarchical cluster of differences between *BRCAX* samples with or without a *BRCA1* or *BRCA2* sequence variant of Low Clinical Significance. Unsupervised cluster analysis was performed using 1778 genes that varied in expression 2-fold from the mean in 10% of *BRCAX* without a *BRCA1/2* LCS variant and *BRCAX* samples with an LCS. The tree structure at the top of the cluster shows how related the samples are to each other. The majority of *BRCAX* samples without an LCS (black) clustered in a distinct group away from *BRCAX* with an LCS variant (red).

The ultimate aim of this experiment was to establish if gene expression profiles could distinguish between *BRCA1* or *BRCA2* pathogenic mutation carriers and familial breast cancer cases whose disease was not attributable to *BRCA1* or *BRCA2* mutations (*BRCAX* cases). *BRCAX* breast cancer families are likely to result from mutations in several other genes, and thus represent a heterogeneous group. Moreover, included in the *BRCAX* group were a subset of 10 *BRCAX* individuals who carried a *BRCA1/2* variant previously classified to be of low clinical significance using multifactorial likelihood approaches [8],[19],[21],[22]. Unsupervised hierarchical clustering showed that *BRCAX* LCLs containing a *BRCA1* or *BRCA2* variant of low clinical significance clustered away from the majority of remaining *BRCAX* samples (Figure 1). A t-test with Benjamini and Hochberg multiple testing correction [34] was performed to determine if there were gene expression differences between the *BRCAX* individuals with an LCS variant and those without an LCS variant. Expression of 631 genes differed between the two *BRCAX* subgroups (5% of the 631 genes identified would be expected to pass this restriction by chance). For this reason, *BRCAX* samples were stratified according to the presence of an LCS variant for further analyses.

Gene expression is similar for carriers of *BRCA1* and *BRCA2* truncating mutations and rare sequence variants of low clinical significance, but differs from *BRCA1* and *BRCA2* missense mutations and *BRCAX* non-*BRCA1/2* familial cases.

**Figure 2:**
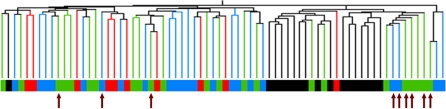
Unsupervised Hierarchical Cluster of Differences between *BRCA1*, *BRCA2* and *BRCAX* samples. Clustering was based on 4751 genes which varied 2-fold difference in gene expression in at least 10% of samples. There are two main clusters, the *BRCAX* samples without a *BRCA1/2* LCS variant (black) cluster to the right, whereas *BRCA1* (green*), BRCA2* (blue*)* and *BRCAX* samples with a *BRCA1/2* LCS variant (red) are predominantly located in the left cluster. The missense pathogenic mutations of *BRCA1* or *BRCA2* are indicated with arrows and 6/9 cluster closest to the *BRCAX* LCLs.

Unsupervised hierarchical clustering (Figure 2) of LCL expression data from all samples revealed that *BRCA1* and *BRCA2* samples were more similar to each other than *BRCAX* samples without an LCS variant. This result suggests that germline effects of heterozygous mutations in *BRCA1* and *BRCA2* cannot easily be separated using the experimental conditions used in this study. Although *BRCAX* samples tended to cluster distinctly from *BRCA1*/2 samples, nine of ten *BRCAX* individuals who carried a *BRCA1/2* variant previously classified to be of low clinical significance fell within the major *BRCA1* or *BRCA2* mutation cluster. In contrast, six of the nine pathogenic missense mutations of *BRCA1* or *BRCA2* fell into a *BRCA1/2* outlier group, which clustered closer to the *BRCAX* samples.

### Gaussian Process Classifier Prediction of *BRCA1*, *BRCA2* and *BRCAX* Mutation Status

To determine the accuracy of using gene expression data from LCLs to predict *BRCA1/2* pathogenic carriers and *BRCAX* individuals, we used a Gaussian Process Classifier (GPC). GPC analysis was used previously in an analysis of microarray profiles from irradiated short-term fibroblasts of *BRCA1/2* mutation carriers [31], and allows for multiway comparison of groups. For GPC analysis 2031 genes which were significantly over/under-expressed at the 5% significance level were selected. The GPC was used in a three way comparison to compare *BRCA1* truncating mutation carriers to *BRCA2* truncating mutation carriers, and to *BRCAX* samples without an LCS variant. Samples with *BRCA1* or *BRCA2* pathogenic missense mutations or classified as *BRCAX* with an LCS variant were then included to determine their affect on the prediction accuracy. A summary of the prediction accuracy is shown in Table 1. The highest prediction accuracy (62.26%) was achieved when the analysis excluded samples classified as *BRCAX* with an LCS, and samples with *BRCA1* or *BRCA2* missense mutations. This prediction accuracy is above the expected performance, as a random prediction with three classes comprised of a similar sample number would be 33% accuracy. When *BRCA1* and *BRCA2* samples were compared to only *BRCAX* samples with an LCS variant, the prediction dropped to 43.46%, and the addition of the *BRCAX* samples without an LCS variant improved the accuracy. In all comparisons the inclusion of the pathogenic non-truncating mutations of *BRCA1* and *BRCA2* lowered the prediction accuracy.

**Table 1:** Accuracy of Prediction of Mutation Status using a Gaussian Process Classifier*

	*BRCA1*	*BRCA1*	*BRCA1*	*BRCA1*
	Vs *BRCA2*	Vs *BRCA2*	Vs *BRCA2*	Vs *BRCA2*
	Vs *BRCAX* (no LCS)	Vs *BRCAX LCS*	Vs *BRCAX* (no LCS) +*BRCAX LCS*	Vs *BRCAX* (no LCS)
				Vs *BRCAX LCS*
Excluding pathogenic missense	**62.26%**	43.46%	52.36%	53.96%
Including pathogenic missense	46.77%	40%	45.83%	47.22%

*: 2031 genes were used to predict mutation status between groups, as described in the methods section.

### Comparison of Gene Expression Profiles between *BRCAX* and *BRCA1* or *BRCA2* LCLs

**Figure 3:**
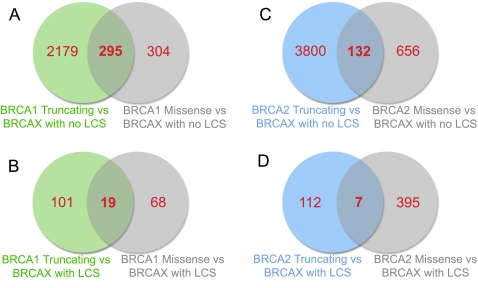
Venn Diagrams of Differences between *BRCA1* or *BRCA2* and *BRCAX* LCLs with or without a LCS. T-tests (p-value<0.05) were performed to determine genes that differed between LCLs as follows: A) *BRCA1* Truncating mutations vs *BRCAX* with no LCS, and *BRCA1* missense mutations vs *BRCAX* with no LCS; B) *BRCA1* Truncating mutations vs *BRCAX* with an LCS, and *BRCA1* missense mutations vs *BRCAX* with an LCS. C) *BRCA2* Truncating mutations vs *BRCAX* with no LCS, and *BRCA2* missense mutations vs *BRCAX* with no LCS; D) *BRCA2* Truncating mutations vs *BRCAX* with an LCS, and *BRCA1* missense mutations vs *BRCAX* with an LCS. For each comparison, the overlap of genes is shown.

In the clinical setting, unclassified sequence variants of *BRCA1* or *BRCA2* are generally identified after full sequencing of both genes. Therefore the most common clinical question is whether a variant in *BRCA1 or BRCA2* is pathogenic or not. We thus performed pair wise analyses to determine if *BRCAX* samples could be distinguished from those with pathogenic mutations in *BRCA1 or BRCA2.* Based on observations from hierarchical clustering analyses and the GPC analysis, we also considered the possibility that the effect of pathogenic *BRCA1/2* mutations (truncating or missense) affected LCL gene expression. T-tests were performed using the 20,874 detected probes to elucidate gene differences between i) *BRCA1* or *BRCA2* truncating mutations vs *BRCAX* without an LCS variant; ii) *BRCA1* or *BRCA2* missense mutations vs *BRCAX* without an LCS variant. The number of genes which passed these restrictions and the overlap between them is outlined in Figure 3A and 3C. The comparisons were then repeated with *BRCAX* with an LCS variant (Figure 3B and 3D). As expected when *BRCA1* and *BRCA2* were compared to *BRCAX* samples without an LCS variant, a greater number of genes were deemed significant compared to *BRCA1* or *BRCA2* vs *BRCAX* samples with an LCS variant.

### Support Vector Machines Prediction of *BRCA1*, *BRCA2* and *BRCAX* Mutation Status

SVM is a widely accepted classification approach for assessing differences in mRNA expression, and was used to compare *BRCA1* or *BRCA2* individually to *BRCAX* samples. Since our detailed analysis of gene lists showed that mutation effect (truncating or missense substitution) appears to affect the genes that are differentially expressed in the carriers after IR (Figure 3), we assessed if these gene differences will affect the predictions. We used SVM with the top 200 genes from the comparison of *BRCA1* or *BRCA2* truncating mutations to *BRCAX*, and the top 200 genes from the comparison of *BRCA1* and *BRCA2* missense mutations to *BRCAX* (Figure 3A and 3C). The genes which differed between *BRCA1* or *BRCA2* and *BRCAX* with an LCS variant were not used in this comparison as too few genes passed the restriction (Figure 3B and 3D). The top 200 genes are listed in Tables S2, S3, S4, and S5 and the overlap of the top 200 genes used for prediction from [*BRCA1* (missense) vs *BRCAX* (noLCS)] and [*BRCA1* (truncating) vs *BRCAX* (noLCS)] was 16 transcripts, with no overlap between the top 200 genes from [*BRCA2* (missense) vs *BRCAX* (noLCS)] and [*BRCA2* (truncating) vs *BRCAX* (noLCS)]. A total of 715 different genes were represented in the four lists of top 200 gene-lists from comparison of *BRCAX* (no LCS) to the different *BRCA1/2* groups above. The results are summarised in Tables 2 and 3. The *BRCA2* truncating pathogenic carriers were consistently predicted with higher accuracy compared to *BRCA1* truncating pathogenic carriers. The accuracy of prediction was improved when the gene list used for prediction was appropriate to the mutation effect (truncating or missense) being tested. When the missense-associated gene list was used, pathogenic truncating mutations were predicted with 35% and 68% accuracy for *BRCA1* and *BRCA2,* respectively. Predictions increased to 71% and 84% for *BRCA1* and *BRCA2,* respectively, using the truncating-associated genes. Similarly, the pathogenic missense mutation carriers were predicted with 83% and 100% accuracy when the missense-associated gene list is used, but this accuracy was lower or remained the same when the truncating-specific gene list was used (83% and 0%). Prediction of *BRCAX* samples that did not carry an LCS variant was high in all comparisons (82–94%). In contrast, prediction of BRCAX samples that did carry an LCS variant was poor (40–50%).

**Table 2:** Mutation Prediction of *BRCA1* and *BRCAX* samples based on SVM

Gene list used for prediction*	Proportion of mutation group correctly predicted			
	*BRCA1* Truncating	*BRCA1* Missense	*BRCAX* (no LCS)	*BRCAX* with an LCS
*BRCA1* Truncating list	12/17 (71%)	5/6 (83%)	16/17 (94%)	5/10 (50%)
*BRCA1* Missense list	6/17 (35%)	5/6 (83%)	14/17 (82%)	4/10 (40%)

*: Lists included the 200 Highest Ranked Genes from the comparison of *BRCA1* to *BRCAX* samples without an LCS variant, as described in the methods.

**Table 3:** Mutation Prediction of *BRCA2* and *BRCAX* samples based on SVM

Gene list used for prediction*	Proportion of mutation group correctly predicted			
	*BRCA2* Truncating	*BRCA2* Missense	*BRCAX* (no LCS)	*BRCAX* with an LCS
*BRCA2* Truncating list	16/19 (84%)	0/3 (0%)	16/17 (94%)	4/10 (40%)
*BRCA2* Missense list	13/19 (68%)	3/3 (100%)	16/17 (94%)	4/10 (40%)

*: Lists included the 200 Highest Ranked Genes from the comparison of *BRCA2* to *BRCAX* samples without an LCS variant, as described in the methods.

**Figure 4:**
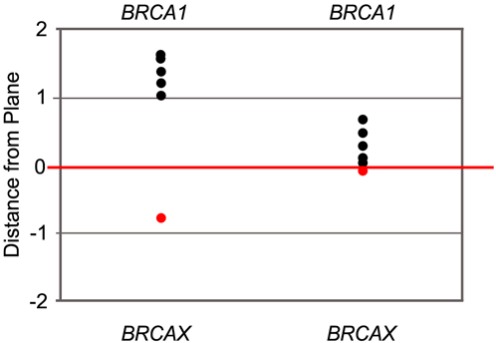
Confidence of Predictions for Missense *BRCA1* LCLs as Determined by Distance from the SVM Plane. The SVM plane separating *BRCA1* from *BRCAX* is shown by the red line. If the sample falls over the line (black point) the missense mutation is correctly predicted as pathogenic for *BRCA1* mutation. If the sample falls under the line (red point) the missense mutation is incorrectly predicted as *BRCAX*. The gene lists used for the predictions are the top 200 genes from *BRCA1* missense vs *BRCAX*, and the top 200 genes from *BRCA1* Truncating vs *BRCAX*.

When using SVM, the significance of the predictions can also be represented by the distance the prediction is from the plane, where predictions called with greater confidence are further from the plane that separates the *BRCA1* (or *BRCA2*) and *BRCAX* samples. The significance of the predictions for the *BRCA1* pathogenic missense mutations is summarised in Figure 4. Although both missense and truncating gene lists correctly predicted 5 of 6 missense mutations, the results show that there is much greater confidence in the 5 correctly predicted missense mutations when using the missense-derived list.

### Pathway Analysis of Genes Associated with Pathogenic Mutations in *BRCA1* or *BRCA2*

Ingenuity Pathway Analysis of genes which differed between the LCLs carrying pathogenic truncating or missense mutations of *BRCA1* or *BRCA2* compared to *BRCAX* samples without an LCS variant was performed to determine the potential functional relevance of the differentially expressed genes. All *BRCA1* and *BRCA2* pathogenic mutations resulted in gene expression changes relating to cell cycle, cancer and cellular growth and development, while *BRCA1* and *BRCA2* missense mutations shared some additional similarities (cell death and cell development pathways). There were also alterations in several pathways that were unique to *BRCA1* truncating mutations, *BRCA2* truncating mutations, *BRCA1* missense mutations, or BRCA2 missense mutations (Figure S1).

## Discussion

It is difficult to counsel patients with a strong family history of breast cancer who are found to carry an unclassified variant in *BRCA1* or *BRCA2*. While management at the level of the family should remain unchanged from that of a *BRCAX* family with no knowledge of a *BRCA1/2* mutation, some individuals from high-risk families may nevertheless interpret information about an unclassified variant to alter their choices regarding prophylactic surgery for example, and so require careful counselling. Gene expression profiling can be used to classify samples based on phenotype, and its frequent use in laboratories world-wide holds great promise for clinical application, to the extent that profiling tools are being developed for diagnostic use e.g. Agendia Inc. (http://www.agendia.com/).

Expression profiles of short-term fibroblasts have previously been reported to separate carriers of a heterozygous mutation in the *BRCA1* or *BRCA2* genes from sporadic breast-cancer-affected controls [30],[31]. We wished to determine if expression profiling of LCLs could similarly be used to predict *BRCA1* or *BRCA2* mutation status, with the ultimate aim of predicting the significance of unclassified variants of *BRCA1* or *BRCA2.* We chose LCLs as a minimally invasive source of germline material that can be maintained as long term cultures, and because previous studies have shown that LCL array profiling is robust to sourcing of LCLs established in different laboratories [33]. We compared expression profiles of irradiated LCLs from *BRCA1* and *BRCA2* carriers to those of non-*BRCA1/2 BRCAX* familial breast cancer patients, an appropriate reference group for the proposed evaluation of unclassified variants identified in familial breast cancer patients. A relatively early time-point of 30 minutes post-irradiation was chosen to capture gene expression initiation, and minimize possible downstream compensation effects. It has previously been shown that 10Gy IR treatment of normal LCLs has an effect on the transcriptional response, with greatest change in mRNA levels for most genes within one hour post-treatment [35], and studies of mouse brain gene expression after whole-body low-dose irradiation have shown that a large number of early IR response genes can be measured at the 30 minute time point [36].

A number of *BRCAX* cases carried *BRCA1* or *BRCA2* sequence variants that had been previously classified using multifactorial likelihood modelling methods to be neutral or of low clinical significance-that is, these rare variants are extremely unlikely to be a high-risk mutation in either of these genes, but the modelling methods used cannot assess whether they are truly neutral or associated with a much lower risk of disease. We found that the *BRCAX* samples with such LCS variants were separated from the majority of *BRCAX* samples without such LCS variants using unsupervised hierarchical clustering. This result indicates that LCS samples differ in expression profile as a result of their *BRCA1* or *BRCA2* sequence variant, and was substantiated by the class prediction methods: GPC prediction of the *BRCAX* samples decreased in accuracy when *BRCAX* samples with an LCS were included. In addition, SVM to detect *BRCA1* or *BRCA2* mutation-related gene lists yielded differences in the significant genes for comparisons to *BRCAX* samples without an LCS variant, compared to *BRCAX* samples with an LCS variant. Accordingly, prediction of *BRCAX* subgroup status based on the more robust gene list derived from comparisons to *BRCAX* individuals without an LCS variant was generally poorer for *BRCAX* samples with an LCS (40–50%) compared to those without an LCS (82%–94%). These rather provocative results indicate that the possible effect of all rare variants should be considered in development of assays to assess which variants have features of high-risk mutations. Moreover, the similarity in expression profile of these variants to other *BRCA1/2* pathogenic mutations suggests that at least some of these LCS variants may confer small-moderate risks of breast cancer, presumably acting in concert with alterations in other genes in the *BRCA1/2* pathway to lead to breast cancer. Given the rarity of these variants, alternative statistical approaches will be required to assess the risk of cancer associated with them.

The assay conditions used in this study could not distinguish between samples with pathogenic *BRCA1* mutations and those with pathogenic *BRCA2* mutations. Ionising radiation has previously been show to separate fibroblast cells which carry *BRCA1* or *BRCA2* mutations from sporadic cases with 100% accuracy [31], but our experiment differs in several respects. We compared *BRCA1* and *BRCA2* cases to familial *BRCAX* cases as an appropriate reference group for familial breast cases likely to be identified as carriers of *BRCA1/2* mutations or unclassified variants, we used LCLs instead of fibroblasts, we selected a lower IR exposure (10Gy vs 15Gy), and we chose a relatively early time point of 30 mins after exposure to IR in order to gain a better understanding of the functional differences in response to IR between the *BRCA1*, *BRCA2* and *BRCAX* cell lines. Some or all of these factors may explain the difference in the ability of this study to distinguish *BRCA1* from *BRCA2*, both of which are involved in DNA damage repair. However, differences in post-irradiation response between *BRCAX* individuals and *BRCA1/2* mutation carriers are supported by alternative analysis we have conducted of the subset of genes reported to be involved in post-irradiation response, comparing mutation-negative normal female controls to *BRCAX* individuals without an LCS variant, or to *BRCA1* or *BRCA2* truncating mutation carriers. Our results indicate substantial differences in radiation response between normal controls and the patient groups, and also considerable differences between the *BRCAX* group and *BRCA1* and *BRCA2* carriers [37]. Alternative IR exposures and/or post-IR timepoints, and possibly different DNA damaging agents, should be considered for future experiments.

The ultimate aim of this experiment was to identify array profiles that would be useful for the classification of unclassified sequence variants of *BRCA1* or *BRCA2*. In the clinical setting, individuals generally present with full sequencing of both genes, and presence of a variant in one gene or the other. We thus assessed the ability to distinguish *BRCA1* or *BRCA2*, separately, from *BRCAX* individuals. Importantly, since most unclassified variants are predicted to cause amino acid substitutions, we also assessed the relevance of mutation effect for expression profiles. We found that the genes which significantly differed between *BRCA1* or *BRCA2* and *BRCAX* LCLs were dependent on mutation effect. Accordingly, the SVM prediction for each mutation effect was best if the appropriate gene list was used, in terms of both accuracy of prediction (*BRCA1* or *BRCA2* vs *BRCAX*) and confidence in the classification as determined by the distance of the prediction from the SVM plane. Thus we strongly urge that mutation effect is taken into account if this type of assay is to be developed for use in predicting the clinical significance of *BRCA1/2* variants. The current challenge is that few missense variants have been classified with respect to their clinical significance, with the only 23 individual missense variants termed clinically important by BIC, 17 in *BRCA1* and six in *BRCA2*. Moreover, these are restricted in terms of the domains/regions in which they occur, residing in the BRCA1 start site (n = 2), ring finger (n = 4) or transactivation domains (n = 11), and the BRCA2 CDK2 phosphorylation site (n = 3) or at one codon (2336, n = 3) in a region of unknown function. It will thus be difficult to accrue a panel of known pathogenic missense variants for use in such predictive assays, and will require a concerted collaborative effort. Assuming sufficient pathogenic variants are identified, the successful execution of such a study may eventually distinguish missense-associated gene expression patterns that are generic to missense mutations, and/or those that are specific to the domain location of missense mutations. In addition, a possibly greater challenge will be identifying assay conditions (cell type, perturbation, time-point etc) that can also identify gene expression differences between patients with rare variants of low clinical significance in *BRCA1* and/or *BRCA2* and those with truly high-risk pathogenic mutations (truncating or missense) in these genes. Our study, using conditions that were not optimal for separating *BRCA1* and *BRCA2* mutations nevertheless identified gene expression differences between *BRCA1/2* pathogenic mutations and LCS variants, suggesting that larger sample sizes and further experimentation may identify a more robust gene list to separate pathogenic mutations, variants of low clinical significance, and individuals with no sequence alterations in *BRCA1/2*.

Pathway analysis confirming altered expression of cancer, cell proliferation and cell cycle pathways in *BRCA1* and *BRCA2* mutation carrier groups is consistent with the known functions of *BRCA1* and *BRCA2* [38],[39]. The pathway differences by mutation type such as cell death and development may reflect that the majority of truncating mutations result in activation of the nonsense mediated decay pathway [40] and complete loss of protein, whereas most missense mutations are likely to result in more subtle effects through ablation of individual functional domains. Some pathways identified were unexpected and are only present in a single mutation type, and it is thus likely that at least some of these pathways were generated by chance alone.

In conclusion, we have provided evidence that carriers of *BRCA1* and *BRCA2* variants considered to be of low clinical significance have array profiles distinct from other non-*BRCA1/2* familial cases, but resembling profiles of pathogenic *BRCA1/2* cases, indicating that further work will be required to evaluate their possible association with a low-moderate risk of cancer. We have also shown that it will be important to consider mutation effect when developing array-based assays for predicting the clinical significance of *BRCA1* or *BRCA2* unclassified variants. Lastly, our findings demonstrate the ability of array profiling of immortalized lines derived from lymphoblastoid cells to detect germline mutations in genes that result in breast and ovarian cancer, and thus have relevance to the investigation of other genetic diseases irrespective of the organs or tissues they affect.

## Materials and Methods

### Subjects and Lymphoblastoid Cell Lines

LCLs were derived from breast cancer-affected women recruited into the Kathleen Cuningham Foundation for Research into Breast Cancer (kConFab), a consortium which ascertains multiple-case breast cancer families [41]. These include families in which one or more carriers of a *BRCA1* or *BRCA2* mutation have been identified, and families in which no predisposing mutation has been identified (*BRCAX*). The recruitment criteria for *BRCAX* families are: 1) at least one member of the family at high-risk according to the National Breast Cancer Centre Category III guidelines (http://www.nbcc.org.au), and four or more cases of breast or ovarian cancer (on one side of the family), and two or more living affecteds with breast or ovarian cancer, and four or more living first or second degree unaffected female relatives of affected cases, over the age of 18 ; 2) two or three cases of breast or ovarian cancer (on one side of the family) in same or adjacent generations, if at least one of these cases is ‘high risk’ (i.e. male breast cancer, bilateral breast cancer, breast plus ovarian cancer in the same individual, or breast cancer with onset less than 40 years), and two or more living affected cases with breast or ovarian cancer, and four or more living first or second degree unaffected female relatives of affected cases, over the age of 18.

Classifications for *BRCA1* and *BRCA2* pathogenic mutations and variants of low clinical significance (LCS) are described on http://www.kconfab.org/Progress/Classification.shtml. Briefly, LCS variants include *BRCA1* or *BRCA2* variants described *in trans* with a deleterious mutation in the same gene in an individual and occur at a frequency of less than 1% in unaffected controls, or considered neutral/low clinical significance as measured using multifactorial likelihood approaches [16],[19],[21],[22].

A cohort of 72 LCLs were used in this study. The full listing of mutation details for LCLs is shown in Table S1. In brief, the study included:

- 23 LCLs from women carrying a pathogenic mutation in *BRCA1*, 17 of which are predicted to lead to a truncated protein, and six of which were missense mutations (2× 300 T>G C61G; 2× 5242 C>A A1708E; 1× 5331 G>A G1738R; 1× 5632 T>A V1838E);
- 22 LCLs from women carrying a pathogenic mutation in *BRCA2*, 19 of which are predicted to lead to a truncated protein, and three of which were missense mutations (3× 8395 G>C D2723H, one of which also carried the LCS variant 9079 G>A A2951T);
- 27 LCLs from women from breast cancer families that have tested negative for pathogenic mutations in *BRCA1* or *BRCA2* (*BRCAX*) after complete sequencing and multiplex ligation-dependent probe amplification gene dosage assay (MLPA) large deletion testing of *BRCA1* and *BRCA2*. Ten samples, carried either *BRCA1* or *BRCA2* sequence germline variants considered from multifactorial likelihood classification to be LCS (*BRCA1* 3582 G>C D1155H, 1605 C>T R496C, 5236 G>C G1706A (2 samples); *BRCA2* 353 A>G Y42C, 2834 C>T S869L, 3031 G>A D935N (3 samples), 8795 A>C E2856A) [16],[19],[21],[22](unpublished data). The remaining 17 samples carried no *BRCA1* or *BRCA2* sequence variants other than common polymorphisms.

### Gene Expression Profiling

LCLs were grown in RPMI 1640 media with 15% fetal bovine serum, 1% penicillin-streptomycin and 1% L-glutamine. The cell number was normalised and fresh medium was added to cells 24hr prior to irradiation with 10Gy, using a calibrated Cs137 c-source delivering 1 Gy/1.5 min. Total RNA was harvested 30min later using an RNeasy kit (Qiagen, Doncaster, VIC). The Illumina Totalprep RNA amplification kit (Ambion, Austin, TX) was used to amplify and biotinylate 450ng of total RNA. Biotinylated RNA was hybridised overnight at 55°C to Illumina Human-6 version 1 BeadChips containing >46,000 probes (Illumina Inc., San Diego, CA). The microarrays were washed, stained with streptavidin-Cy3, and then scanned with an Illumina BeadArray Scanner. Duplicate arrays were performed for eight cell lines across the different groups for quality control purposes, with duplicates performed on different days. All duplicate arrays showed highest correlation with each other (correlation >0.98). Duplicate samples were not included in analysis. Comparative real-time PCR was performed for ten genes on 6–8 samples, using GAPDH to normalise all data, and the comparative cycle threshold method for analysis. Paired student *t* tests were performed to determine the significance of gene expression changes. Expression differences were validated for 8/10 genes tested.

### Data Analysis

Raw data was imported into Illumina Beadstudio and then exported into Genespring v7.3 (Agilent Technologies, Forest Hill, VIC) for further analysis. Data was normalised (per chip normalized to 50th percentile and per gene normalized to median) and filtered using an Illumina detection score of >0.99 in at least one sample, which yielded 20,874 probes that were used in all further analyses. The majority of these probes used in the analysis were designed by Illumina to assay the curated portion of the NIH Ref sequence database-16,923 were present in the Ref sequence database, comprising 65% of all Ref sequence-listed probes on the array. Transcripts which had a >2-fold change versus the mean were visualised using unsupervised Hierarchical Clustering (Figures 1 and 2). The clustering method used was a Pearson correlation similarity measure with an average linkage clustering algorithm. Two different methods were used to classify LCLs based on mutation status: (1) A multi comparison Gaussian Process Classifier (GPC) [42] with Leave-One-Out cross-validation to determine the prediction errors, as previously used to predict *BRCA1/BRCA2* mutation status of irradiated fibroblasts [31]; (2) A linear classification method commonly used for classification of microarray data, Support Vector Machines (SVM) [43] with Leave-One-Out cross validation. The GPC analysis used 2031 genes which were derived from a t-test to select the genes that were significantly over/under-expressed at the 5% significance, while the SVM used genes from the 20,874 detected probes which differed between groups of LCLs using a t-test p of 0.05. All resulting gene lists are available as supplementary data and all data is available via GEO: Accession number GSE10905.

Ingenuity Pathway Analysis (Ingenuity Systems, www.ingenuity.com) was used for biological interpretation of gene lists. Analysis of the transcripts found to be up- and down-regulated in irradiated LCLs as identified for the different mutation categories identified those biochemical networks most likely to be affected by a *BRCA1* and *BRCA2* truncating and missense mutation, relative to *BRCAX*. Those pathways with multiple hits or a significance score ≥20 were then compared.

## Supporting Information

Figure S1 Biological Pathways defined by genes dysregulated in *BRCA1* and *BRCA2* mutation carriers. Pathways identified by Ingenuity pathway analysis of the top 200 genes defined for truncating and missense *BRCA1* or *BRCA2* mutations compared to BRCAX without an LCS were compared for overlap. Bold lines and pathways denoted in uppercase indicate biological pathways identified as differentially expressed in both BRCA1 and BRCA2
(0.20 MB TIF)

Table S1 Detailed Mutation Status of LCLs.
(0.03 MB XLS)

Table S2 The top 200 significant genes from the comparison of *BRCA1* Missense vs *BRCAX* without an LCS.
(0.05 MB XLS)

Table S3 The top 200 significant genes from the comparison of *BRCA1* Truncating vs *BRCAX* without an LCS.
(0.05 MB XLS)

Table S4 The top 200 significant genes from the comparison of *BRCA2* Missense vs *BRCAX* without an LCS.
(0.06 MB XLS)

Table S5 The top 200 significant genes from the comparison of *BRCA2* Truncating vs *BRCAX* without an LCS.
(0.05 MB XLS)
