# Supplementary figures and images for: BRCA1 and BRCA2 Missense Variants of High and Low Clinical Significance Influence Lymphoblastoid Cell Line Post-Irradiation Gene Expression
Source: PLoS Genet. 2008 May 23;4(5):e1000080. doi: 10.1371/journal.pgen.1000080 (PMC2375115; doi:10.1371/journal.pgen.1000080)

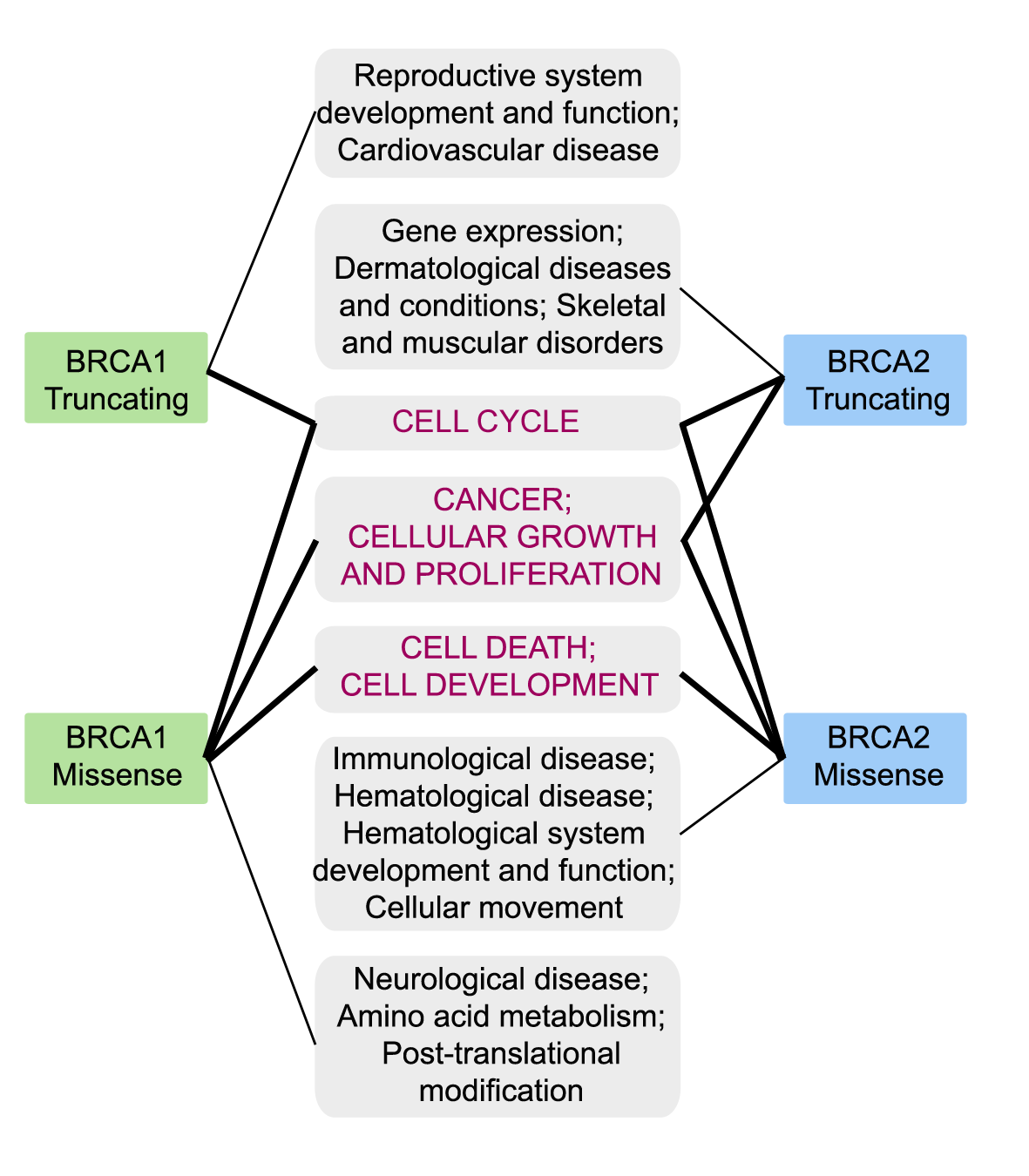

Supplement: Figure S1 — Biological Pathways defined by genes dysregulated in BRCA1 and BRCA2 mutation carriers. Pathways identified by Ingenuity pathway analysis of the top 200 genes defined for truncating and missense BRCA1 or BRCA2 mutations compared to BRCAX without an LCS were compared for overlap. Bold lines and pathways denoted in uppercase indicate biological pathways identified as differentially expressed in both BRCA1 and BRCA2 (0.20 MB TIF) [file pgen.1000080.s001.tif]
